# Pregnancy in May–Hegglin Anomaly: Diagnostic Challenges and Management Considerations

**DOI:** 10.1155/crh/4997232

**Published:** 2025-07-10

**Authors:** Metban Mastanzade, Alper Koç

**Affiliations:** ^1^Division of Hematology, Department of Internal Medicine, Istanbul University Istanbul Faculty of Medicine, Istanbul, Turkey; ^2^Division of Hematology, Department of Internal Medicine, Elazığ Fethi Sekin City Hospital, Elazığ, Turkey

**Keywords:** cesarean delivery, Döhle-like inclusions, May–Hegglin anomaly, MYH9 gene, prenatal care, thrombocytopenia

## Abstract

**Introduction:** May–Hegglin anomaly (MHA) is a rare autosomal dominant genetic disorder caused by mutations in the MYH9 gene, leading to the presence of Döhle-like inclusions in neutrophils, macrothrombocytes, and thrombocytopenia. This report presents a unique case of a 33-year-old pregnant woman diagnosed with MHA and discusses the diagnostic challenges and management strategies.

**Case Presentation:** A 33-year-old pregnant woman, 17 weeks into her pregnancy, presented with a history of persistent thrombocytopenia. She had previously been diagnosed with immune thrombocytopenia (ITP) and treated with steroids, intravenous immunoglobulin (IVIG), and thrombopoietin receptor agonists (TPO-RA). Her platelet counts had been between 35,000 and 50,000/μL. Upon referral to the hematology clinic, her platelet count was critically low at 15,000/μL, but the mean platelet volume (MPV) remained within normal limits. Despite her low platelet count, her coagulation profile was normal, and physical examination showed no pathological findings.

**Diagnostic Assessment:** The patient's blood smear revealed giant platelets and Döhle-like inclusions in the granulocytes. Genetic testing confirmed a heterozygous mutation in the MYH9 gene, leading to the diagnosis of MHA.

**Therapeutic Intervention:** Due to the risks associated with thrombocytopenia in pregnancy, her prenatal care included routine platelet monitoring and a normal bleeding time assessment. The patient underwent a cesarean delivery under general anesthesia, which resulted in the birth of a healthy baby boy.

**Conclusion:** The case highlights the importance of accurate diagnosis and careful monitoring in managing pregnancy in patients with MHA. A multidisciplinary approach involving obstetricians and hematologists is crucial for optimizing maternal and neonatal outcomes.

## 1. Introduction

May–Hegglin anomaly (MHA) is a rare autosomal dominant genetic disorder characterized by distinct hematological abnormalities. The condition arises from mutations in the MYH9 gene, which encodes nonmuscle myosin heavy chain IIA, an essential protein involved in various cellular processes including cytoskeletal organization and cell motility [1]. MYH9 gene mutations disrupt the normal function of this protein, leading to a constellation of clinical features [2].

Clinically, MHA is marked by the presence of abnormal cytoplasmic inclusions in neutrophils known as Döhle-like bodies, which are thought to be aggregates of abnormal myosin. Inclusions that resemble Dohle bodies are seen not only in neutrophils but also in other white blood cells such as monocytes, eosinophils, and basophils [3]. Under Wright–Giemsa stain, they appear as large, spindle-shaped, and pale blue structures. These inclusions do not show up in platelets. Instead, platelets are often larger and can appear in larger or giant forms. This presence of macrothrombocytes in some patients can sometimes cause automated analyzers to underestimate the platelet count.

The phenotypic presentation of MHA can overlap with other myosin-related disorders and platelet abnormalities, such as May–Hegglin-like conditions and certain forms of inherited thrombocytopenia. Therefore, a comprehensive diagnostic approach is necessary to distinguish MHA from other hematological disorders. This often involves detailed clinical assessment, peripheral blood smear analysis, and confirmation through genetic testing [2].

The management of MHA, particularly in the context of pregnancy, necessitates a multidisciplinary approach due to potential complications such as impaired clot formation and associated bleeding risks. Pregnancy in patients with MHA presents unique challenges. While the disorder itself may not directly affect pregnancy outcomes, associated thrombocytopenia and potential clotting issues require careful monitoring [4]. Routine prenatal assessments, including bleeding time and platelet counts, are critical for ensuring both maternal and fetal wellbeing. Moreover, the timing and mode of delivery must be strategically planned to mitigate risks associated with thrombocytopenia and other hematological abnormalities [5].

This case report aims to illustrate the clinical presentation, diagnostic approach, and management considerations for a patient with MHA during pregnancy. By presenting this case, we highlight the importance of accurate diagnosis and tailored management strategies, emphasizing the need for an integrated care approach involving hematologists and obstetricians.

## 2. Case Report

A 33-year-old female patient, 17 weeks pregnant, was initially diagnosed with immune thrombocytopenia (ITP). She had been treated with corticosteroids, intravenous immunoglobulin (IVIG), and thrombopoietin receptor agonists (TPO-RA) 3 years prior. Despite these treatments, her platelet counts remained persistently low, ranging between 35,000 and 50,000/μL, indicating no significant or durable therapeutic response. Since that time, she had remained under regular hematological follow-up for persistent thrombocytopenia. Due to persistent thrombocytopenia, she was referred to our hematology clinic by her obstetrician. Upon admission, her platelet count was critically low at 15,000/μL, and mean platelet volume (MPV) was measured at 10.5 fL (normal reference range: 7.5–11.5 fL). The coagulation profile, including prothrombin time, activated partial thromboplastin time, and fibrinogen levels, was entirely within normal limits. The patient denied any prior history of mucosal bleeding, easy bruising, menorrhagia, or other bleeding diatheses.

The patient's physical examination was unremarkable. Blood pressures were within normal limits, and there were no clinical signs of bleeding, petechiae, ecchymosis, or any other complications. Routine biochemical parameters revealed no significant abnormalities.

**Figure 1:**
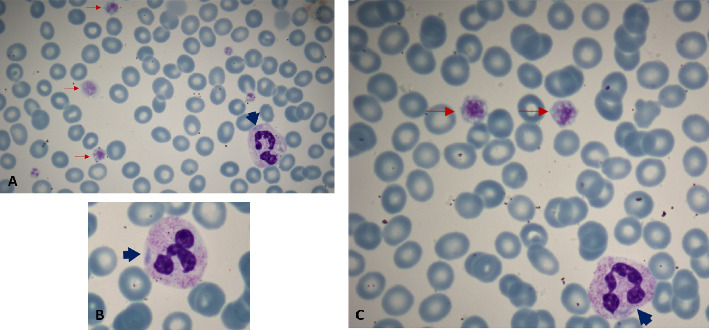
(A–C) Blood smear findings include Döhle-like bodies inside leukocytes and the presence of giant platelets (red arrows: giant platelets and blue arrows: Döhle-like bodies).

The patient's blood smear revealed the presence of giant platelets, estimated to be around 80,000/μL, and Döhle-like inclusions in the cytoplasm of granulocytes (Figures 1(A), 1(B), and 1(C)). The peripheral blood smear was prepared manually using capillary blood obtained via fingertip puncture. The smear was immediately performed at bedside using the wedge (push-slide) technique to minimize platelet aggregation and optimize morphological assessment of large platelets. The slide was air-dried and stained manually with Wright-Giemsa stain following standard laboratory protocols. The mild bluish hue observed in red blood cells is attributed to minor variations in smear thickness and staining properties, which may occasionally result in a pale basophilic background.

A PCR analysis followed was performed to evaluate the MYH9 gene. This analysis identified a heterozygous c.5797C > T mutation, resulting in a nonsense substitution (Arg1933Ter), which introduces a premature stop codon at residue 1933 and leads to the production of a truncated, nonfunctional myosin IIA heavy chain protein. This truncation occurs within the highly conserved nonhelical tail domain, which plays a critical role in filament assembly and cytoskeletal organization. The absence of this region may impair normal myosin filament formation, contributing to the development of macrothrombocytopenia and inclusion body formation characteristic of MHA. Subsequent genetic testing of both parents revealed no evidence of the mutation, indicating that this patient represents a de novo case and is the index case within her family.

Despite the hematological abnormalities, the pregnancy progressed without complications. Given the potential bleeding risks not only due to thrombocytopenia but also to qualitative platelet dysfunction and other hemostatic abnormalities that may be present in patients with MHA, prenatal assessment included IVY bleeding time. The test was performed using a standardized technique with a blood pressure cuff inflated to 40 mmHg and dual incisions made on the forearm using a Surgicutt device. Bleeding time was recorded as 2 min and 15 s (normal range: 2–9 min) [6], indicating adequate primary hemostasis. The patient underwent a cesarean delivery under general anesthesia. At the time of cesarean delivery, the automated platelet count was 20,000/μL. However, due to the presence of giant platelets, which are often underestimated by automated counters, a manual review of the peripheral blood smear estimated the actual platelet count to be approximately 80,000–90,000/μL. Considering the degree of thrombocytopenia, spinal or epidural anesthesia was contraindicated, and general anesthesia was administered. Neither preoperative platelet transfusions nor antifibrinolytic agents were administered. No bleeding complications were observed during or after the procedure, which resulted in the delivery of a healthy baby boy weighing 3100 g. The newborn's platelet count and MPV were within the normal range, and peripheral blood smear demonstrated normally shaped and granulated platelets.

## 3. Discussion

In adults, the predominant etiology of thrombocytopenia is ITP. ITP is an autoimmune disorder characterized by the production of autoantibodies that specifically target and facilitate the destruction of platelets [7]. The condition can be classified as primary, where it arises in the absence of any identifiable secondary cause, or secondary, which is associated with underlying conditions such as systemic lupus erythematosus, chronic viral infections (e.g., HIV or hepatitis C), or drug-induced reactions. Diagnosis of ITP is achieved through exclusion of other causes of thrombocytopenia via comprehensive clinical assessment and diagnostic testing [8]. When careful monitoring and thorough investigation are not conducted, a group of rare diseases that require particular attention and precision in diagnosis may be mistakenly treated as if they were ITP patients.

The diagnosis of MHA can be challenging due to its overlapping features with several inherited and acquired hematologic disorders. In our patient, the presence of persistent thrombocytopenia, macrothrombocytes on peripheral smear, and Döhle-like inclusions initially raised suspicion for alternative causes; however, the absence of additional systemic features commonly seen in other MYH9-related disorders—such as nephropathy, sensorineural hearing loss, or cataracts—may have contributed to the initial misclassification as ITP, leading to an empiric ITP-directed treatment approach. This overlap highlights the potential for diagnostic delay in MHA when typical syndromic features are absent [1]. Ultimately, genetic testing confirmed a heterozygous c.5797C > T (Arg1933Ter) mutation in the MYH9 gene, establishing the definitive diagnosis. This case emphasizes the importance of detailed peripheral smear review and molecular analysis in patients with atypical or refractory thrombocytopenia [9].

Peripheral blood smear analysis is critical for identifying the presence of giant platelets and Döhle-like inclusions. In our patient's peripheral blood smear, Döhle-like inclusions were clearly observed within granulocytes. These inclusions appeared as large, sharply demarcated, spindle-shaped pale blue structures. Unlike Döhle bodies seen in toxic states, which are typically smaller, have blurred margins, and predominantly localize at the periphery of neutrophils, the inclusions in our case were distributed throughout the cytoplasm without strict peripheral predilection [10]. No toxic granulation or other reactive features were observed. These morphological distinctions are consistent with MYH9-related disorders. Despite these findings, a definitive diagnosis requires genetic testing. The identification of MYH9 gene mutations through polymerase chain reaction (PCR) is essential for confirming MHA [1]. In this case, the mutation c.5797C > T was identified, resulting in the Arg1933Ter substitution and a premature stop codon. This mutation in exon 40 causes the R1933X protein alteration [11]. The R1933X missense mutation is located in the 35–43 residue nonhelical end piece, a unique region that distinguishes sarcomeric and nonmuscle class II myosins. As a consequence, the R1933X mutation may disrupt filament assembly, leading to the formation of nondimeric structures that aggregate into paracrystalline arrays resembling “Döhle-like” inclusions [12, 13]. The clinical findings of this patient were milder, similar to those reported in the literature, due to this mutation in the 40^th^ exon of this 41-exon protein-coding gene [14].

Thrombocytopenia is common during pregnancy, with the most common causes being gestational thrombocytopenia, preeclampsia, and immune thrombocytopenia. MHA is a rare cause of thrombocytopenia in pregnancy. Some women are diagnosed before pregnancy, while others are first identified during pregnancy through routine blood tests showing thrombocytopenia. In a review, it was noted that 11 out of 40 women with MHA were diagnosed during pregnancy, similar to our case [4]. These cases are often initially misdiagnosed as immune thrombocytopenia, with no response to treatment. Early and accurate diagnosis of MHA during pregnancy is crucial for the best maternal and neonatal outcomes.

The management of a pregnant patient with MHA involves several unique challenges. First and foremost, the presence of thrombocytopenia and thrombocyte dysfunction in MHA increases the risk of bleeding complications, which requires careful monitoring throughout pregnancy. Although the patient in this case had a normal prenatal IVY bleeding time, which was reassuring, the inherent unpredictability of thrombocytopenia means that constant vigilance is required.

The decision regarding the mode of delivery is particularly critical. There are publications in the literature regarding successful vaginal deliveries [15]. However, after discussing with the patient, we decided on a C-section under general anesthesia. In this case, a C-section was chosen to provide a controlled delivery environment under general anesthesia, which helps manage potential complications associated with low platelet counts [16].

A multidisciplinary approach is fundamental in managing MHA, especially in the context of pregnancy. Effective collaboration between hematologists, obstetricians, and anesthesiologists ensures that all aspects of patient care are addressed comprehensively. This approach facilitates coordinated planning for delivery, including considerations for anesthesia and postdelivery care, and ensures prompt management of any complications.

## 4. Conclusion

This case report highlights the importance of thorough hematological evaluation in patients with low and giant platelets. Identifying Döhle-like inclusions and confirming MYH9 gene mutations through genetic testing are critical for accurate diagnosis. In cases of pregnancy, a collaborative approach involving obstetricians and hematologists ensures comprehensive care and optimal outcomes for both the mother and the newborn. Each patient with MHA should be managed individually, taking into account the specific challenges and potential risks associated with the condition.

## Data Availability

The data that support the findings of this study are available from the corresponding author upon reasonable request.

## References

[1] Feroz W., Park B. S., Siripurapu M. (2024). Non-Muscle Myosin II A: Friend or Foe in Cancer?. *International Journal of Molecular Sciences*.

[2] Fatima S. (2012). May Hegglin Anomaly: Rare Entity with Review of Literature. *Indian Journal of Hematology and Blood Transfusion*.

[3] Untanu R. V., Vajpayee N. (2025). May-Hegglin Anomaly. *StatPearls*.

[4] Hussein B. A., Gomez K., Kadir R. A. (2013). May-Hegglin Anomaly and Pregnancy: A Systematic Review. *Blood Coagulation and Fibrinolysis*.

[5] Yamashita Y., Matsuura R., Kunishima S. (2016). Perinatal Management for a Pregnant Woman With an MYH9 Disorder. *Case Reports in Obstetrics and Gynecology*.

[6] Mielke C. H., Kaneshiro M. M., Maher I. A., Weiner J. M., Rapaport S. I. (1969). The Standardized Normal Ivy Bleeding Time and its Prolongation by Aspirin. *Blood*.

[7] Rodeghiero F., Stasi R., Gernsheimer T. (2009). Standardization of Terminology, Definitions and Outcome Criteria in Immune Thrombocytopenic Purpura of Adults and Children: Report From an International Working Group. *Blood*.

[8] Bu S., Liu M., Yang L. (2025). The Function of T Cells in Immune Thrombocytopenia. *Frontiers in Immunology*.

[9] Thurlapati A., Guntupalli S., Mansour R. (2021). Myosin Heavy Chain 9 (MYH9)-Related Congenital Macrothrombocytopenia. *Cureus*.

[10] Cheng W., Zhao J., Dai W., Xu Y., Liu J. (2023). Light Blue Inclusion Bodies in Neutrophils, Eosinophils, Basophils, Monocytes, and Macrothrombocytopaenia Suggestive of MYH9-RD. *The International Journal of Literary Humanities*.

[11] Syndrome Consortium T. M. H., Cusano R., Gangarossa S. (2000). Mutations in MYH9 Result in the May-Hegglin Anomaly, and Fechtner and Sebastian Syndromes. *Nature Genetics*.

[12] Martignetti J. A., Heath K. E., Harris J. (2000). The Gene for May-Hegglin Anomaly Localizes to a < 1-Mb Region on Chromosome 22q12.3-13.1. *The American Journal of Human Genetics*.

[13] Noris P., Spedini P., Belletti S., Magrini U., Balduini C. L. (1998). Thrombocytopenia, Giant Platelets, and Leukocyte Inclusion Bodies (May-Hegglin Anomaly): Clinical and Laboratory Findings. *The American Journal of Medicine*.

[14] Hodge T. P., Cross R., Kendrick-Jones J. (1992). Role of the COOH-Terminal Nonhelical Tailpiece in the Assembly of a Vertebrate Nonmuscle Myosin Rod. *The Journal of Cell Biology*.

[15] Bizzaro N. (1999). May-Hegglin Anomaly and Uncomplicated Vaginal Delivery: a Report of 41 Cases. *American Journal of Obstetrics and Gynecology*.

[16] Takabayashi R., Nishikido O., Nagano K., Doi A., Nishisako R., Tateda T. (2007). Anesthetic Management for Cesarean Delivery in a Patient with May-Hegglin Anomaly. *Masui*.

